# Impacts on and Care of Psychiatric Patients during the Outbreak of COVID-19

**DOI:** 10.2174/1745017902117010052

**Published:** 2021-07-12

**Authors:** Pavarud Puangsri, Vinn Jinanarong, Apichai Wattanapisit

**Affiliations:** 1 School of Medicine, Walailak University, Thasala, Nakhon Si Thammarat, Thailand; 2 Walailak University Hospital, Thasala, Nakhon Si Thammarat, Thailand; 3 Prachuap Khiri Khan Hospital, Prachuap Khiri Khan, Thailand

**Keywords:** COVID-19, Mental disorders, Mental health services, Patient care, Psychological stress, Case management

## Abstract

The outbreak of coronavirus disease (COVID-19) in December 2019 has led to massive lifestyle, economic, and health changes. The COVID-19 pandemic has had broad impacts on psychiatric patients, exacerbating symptoms such as psychosis, depression, and suicidal ideation. Therefore, we aimed to review the psychological impacts of COVID-19 on psychiatric patients and mental healthcare staff and provide practical guidance for medical staff and authorities.

The main findings of this review included the impacts of COVID-19 on psychiatric patients and mental health professionals as well as the transformation of mental health care. Greater consideration should be given to the care of patients with psychosis and depression because of their lack of self-care ability, neurocognitive impairment, and impaired immune function. Depressive symptoms can be exacerbated due to several factors, such as economic crises, social isolation, and limited physical activity. Unemployment and financial problems can lead to an increased suicide rate. Consequently, mental healthcare workers’ workload can increase, which could lead to burnout and psychological symptoms such as insomnia, depression, and anxiety.

A transformation of psychiatric care is needed during the time of the pandemic. While emergency care should be maintained, outpatient care should be limited to decrease viral spread. Shifting care to telemedicine and community-based psychiatry can be helpful. Inpatient services should be adapted by tightening admission criteria, shortening the length of hospital stays, suspending some group activities, limiting visitors, and preparing for quarantine if necessary. Mental healthcare workers can be supported with telecommunication, appropriate work shifts, alternative accommodations, and good communication between the team leader and staff.

## 1 INTRODUCTION

Prior experiences with coronavirus outbreaks such as MERS-CoV and SARS-CoV contributed to mental health consequences among patients, health care providers, and the general population. Mental problems such as psychophysical stress, posttraumatic stress disorder (PTSD) symptoms, anxiety, depression, and insomnia were common [1-7]. Recent evidence shows the effects of the COVID-19 pandemic on several psychological aspects, such as insomnia, anxiety, and depression, among general populations, patients with psychiatric problems, and healthcare providers [7-10]. Self-quarantine can lead to depression and/or PTSD afterward. Survivors of these CoV infections could develop long-term psychiatric consequences such as depression, anxiety, and memory problems [11]. Moreover, healthcare professionals are affected by COVID-19, both physically and mentally. They are at risk of mental health problems such as depression anxiety and insomnia [12].

Psychiatric patients are more vulnerable to crises than general populations [13, 14]. These patients may be at risk of worsening depression, anxiety, and psychotic symptoms during a crisis [15]. Moreover, psychiatric patients’ higher risk of the inappropriate use of self-medications, nonprescribed medications, and substances *e.g*., alcohol, illegal drugs must be recognized. Such inappropriate use can worsen symptoms and cause a vicious cycle of illness [16-18]. Depressive, psychotic, and other psychiatric patients may be vulnerable to infection due to their health behaviors as well as their physical and mental conditions [19, 20]. Specifically, psychiatric inpatient settings, which have unique characteristics of patient care, involve a high likelihood of transmission due to patient-patient and patient-provider contact [21].

Due to the COVID-19 pandemic and its impacts on several dimensions, there is a need to adapt the care of patients with mental problems. This article aims to present perspectives on the care of psychiatric patients during the outbreak of COVID-19 based on public health concerns and recent literature. The article covers three areas of interest: (i) impacts of COVID-19 on psychiatric patients; (ii) impacts of COVID-19 on mental health professionals; and (iii) the transformation of care and management.

## 2 METHODS

A literature search on PubMed was conducted between April and May 2020. An additional search was performed in February 2021 during the manuscript revision. The review focused on (i) impacts of COVID-19 on psychiatric patients, (ii) impacts of COVID-19 on health care professionals, and (iii) transformation of care and management.

## 3 IMPACTS OF COVID-19 ON PSYCHIATRIC PATIENTS

### 3.1 Psychosis

#### 3.1.1 Direct Impacts of COVID-19

Patients with psychotic symptoms have a higher prevalence of immune reactivity for human coronavirus HKU1 [HCoV-HKU1] and human coronavirus NL63 (HCoV-NL63). The immune reactivity can cause psychotic symptoms related to inflammatory tissue damage as confirmed by autopsy studies. Neurological sequelae such as neuroinflammatory signs, reactive gliosis, astrocytosis, and microglia activation were found in patients with COVID-19 [22, 23]. These findings suggest that neuropsychiatric manifestations can be a direct impact of human coronavirus exposure. However, the role of COVID-19 in the etiopathogenesis of psychiatric diseases needs to be studied further.

#### 3.1.2 Indirect Impacts of COVID-19

Limiting the number of patient visits at the hospital may help decrease viral spread, but on the other hand, it will cause loss to follow-up among patients who lack insight, especially if a tracking system is not available. Patients with psychosis may be among the group of patients who might avoid the treatment setting because of their poor insight, denial of their symptoms, lack of cooperation with staff and their family, and unwillingness to receive the treatment [24-26].

Patients with psychosis may experience adverse consequences due to societal and lifestyle changes, such as social distancing and fear of infection. Abrupt changes in the environment during the pandemic may create many stressful life events *e.g*., social isolation, unemployment, physical inactivity, and physical illness. Eventually, psychotic symptoms can be exacerbated because of these adverse environmental factors [27, 28]. The other mental dimension that is affected by the pandemic among patients with psychosis is mood and behavior. Patients with more severe psychosis will develop more affective and behavioral symptoms *e.g.*, paranoia associated with anger and violence, guilty delusions associated with depression and suicide [29]. During the SARS outbreak, a large amount of evidence of suicide attempts by people with psychotic was found [30]. The physical dimension is also important among patients with psychosis. These patients may susceptible to infection. They can also become spreaders who transmit the disease to others due to their neurocognitive impairment *e.g.*, lack of self-care, slowed perception, impaired problem-solving, reduced planning skills, and poor social cognition [19, 31-33].

### 3.2 Depression

The COVID-19 outbreak has resulted in people losing their opportunities at work and losing income due to a protracted economic crisis. Such a situation increases people’s stress and forces them to ultimately alter their lifestyles. The rapid transformation of the environment (*e.g*., stressful environment, disasters) involves a high risk of depression [34, 35].

Someone who might have been exposed to COVID-19 must self-quarantine for at least 14 days. This self-quarantine keeps them away from their social networks (*e.g*., friends, partners, children, and family). During the quarantine, many activities are limited (*e.g*., shopping at stores, having dinner at restaurants, and playing almost all types of sports) Therefore, quarantined individuals are more likely to develop depression than others. Depression can occur because social distancing or self-quarantine limits people’s activity [20, 36]. Thus, the more physical inactivity, the more depressed a person becomes [37]. Depression can also decrease physical activity [38, 39]. This vicious cycle of depression and a lack of physical activities can be endless.

COVID-19 can also affect patients with depression in many ways. Suffering due to social isolation, anxiety about economic problems, and fear about the future are some of the issues that patients must deal with [40]. This stressful situation can aggravate depressive symptoms in patients. Facing significant, long-term problems that are difficult to solve can lead patients to commit suicide [41, 42].

Patients with major depression can be more prone to viral infection than people without major depression [20]. First, chronic stress directly suppresses immunological function, which places the depressed person at risk of infection [43]. Second, depression can cause deficits in neurocognitive function, such as poor decision-making, that may lead to inadequate self-protection [44].

### 3.3 Anxiety

Anxiety is one of the common mental health problems worldwide. Some studies have found that the prevalence of anxiety is higher during the COVID-19 pandemic. The prevalence of anxiety was around 35% [45]. Results of a survey have reported that 28.8% of people had moderate to severe anxiety [46].

Media misinformation might increase the fear of contagion and infection among the people and sometimes discrimination against the particular community. People who overexpose to social media during the pandemic were more likely to develop anxiety symptoms compared to those who kept using social media as it was before the pandemic [47]. A case report on a patient with panic disorder revealed that the panic episode was precipitated by repeatedly hearing news of the coronavirus infection outbreak [48].

Overestimation of the chance of viral infection, excessive adoption of universal precaution, increased demand for medical services, and global economic concerns can cause a feeling of anxiety [49, 50].

### 3.4 Suicide

Previous pandemics *e.g*., influenza, SARS have resulted in an increased rate of death by suicide [30, 51-53]. Death by suicide may also be increasing during the current pandemic. An increased risk of suicide has been observed during the COVID-19 outbreak [54]. This observation indicates the importance of determining the link between the COVID-19 outbreak and the risk of suicide. In addition, a greater understanding of how suicide occurs is necessary.

Regardless of the COVID-19 crisis, unemployment is also a risk factor for suicide [55]. Currently, the COVID-19 pandemic having a substantial influence on the world economy, causing an economic slowdown. A worsening economy is often associated with unemployment. Some companies and enterprises in the industrial section have shut down or made adjustments by laying off employees to reduce their expenses. Many people have lost their job, and some of them cannot cope with their financial problems. As the unemployment rate increases due to the impact of COVID-19, the suicide attempt rate will also increase [56-58].

Other factors associated with suicide include stressful life events, social change, social rejection, living alone, economic recession, and financial problems [59-61]. These factors seem to be a common problem affecting everyone during the COVID-19 pandemic [62]. The impact of the pandemic affects the general population and especially vulnerable people. There are reports that psychiatric patients developed moderate to severe degrees of suicidal ideation that have led to suicide during the spread of COVID-19 [17, 63]. It has been observed that even patients who were receiving COVID-19 treatment and were quarantined in the hospital committed suicide [64].

## 4 IMPACTS OF COVID-19 ON MENTAL HEALTH PROFESSIONALS

The whole population across the globe has experienced the psychological impacts of COVID-19, including health care professionals. Insomnia was found to be one of the most common symptoms in healthcare professionals, followed by depression and anxiety [9]. However, anxiety was detected at the start of the pandemic, while depression appeared as the outbreak began to subside [65]. Posttraumatic stress disorder is serious psychological distress that has occurred in professionals during and after the outbreak [66]. Healthcare workers working in an area, profoundly impacted by the epidemic, reported more severe psychiatric symptoms than health care workers from the other areas [67]. The severity of mental symptoms was influenced by age, gender, occupation, specialization, type of activities performed, and proximity to COVID-19 patients [68].

Psychiatrists and mental healthcare service providers have a high risk for burnout because they have higher work-related emotional exhaustion than other healthcare workers [69]. People who are drawn toward psychiatry as a career may also have personality characteristics that predispose them to stress, such as neuroticism [70].

A higher suicide rate during the COVID-19 pandemic could lead to more mental health workers exposed to suicide patients. Such exposure could have several effects on mental health workers, such as poor sleep, low mood and anhedonia, preoccupation with suicide, and decreased self-confidence [71].

The implementation of measures to reduce viral spread by psychiatric hospitals, such as strict admission criteria and the use of telemedicine, can be perceived as lowering the standard of care and leading to the moral injury of mental health care workers [72].

As mentioned above, the COVID-19 crisis can exacerbate many psychiatric symptoms. This will eventually increase the workload of psychiatric emergency services [73]. Apart from taking care of psychiatric patients, mental health care workers also have to take responsibility for taking care of other staff who deal with considerable stress during the pandemic [74]. Some countries have established crisis psychological intervention teams to help people deal with stress. The establishment of these teams has reduced the workforce in psychiatric hospitals [21]. All of this could lead to the work overload and burnout of mental health care workers.

## 5 TRANSFORMATION OF CARE AND MANAGEMENT

The number of patients receiving psychiatric treatment worldwide was increasing before the COVID-19 outbreak. Psychiatric outpatient clinics usually have waiting lists for patients with mental illness to receive treatment by psychiatrists [75-79]. Psychiatric institutions should adopt a variety of services to provide continuous care during the crisis. There are five units that can benefit from the adaptation (1); emergency care (2); the outpatient setting (3); the inpatient setting and residential facilities (4); community psychiatry; and (5) mental health workers.

### 5.1 Emergency Care

Patients who present with suicidality, delirium, acute psychosis, severe substance intoxication, and severe substance withdrawal are at risk of life-threatening conditions and serious functional impairment. Immediate care management must be available for these patients [80]. An emergency physician can consult a psychiatrist *via* telephone to reduce chaos in the emergency room. If a psychiatric evaluation is necessary, the psychiatrist should practice physical distancing (*e.g*., sit in another room and video conference) when evaluating or interviewing patients [81].

Healthcare workers in emergency care should be aware of the neuropsychiatric symptoms (*e.g*., agitation, disorganization, psychosis, and alteration of consciousness) of COVID-19 disease. All patients in emergency departments and patients with those symptoms should be evaluated for COVID-19 [82, 83].

### 5.2 Outpatient Setting

The number of visits per day should be limited to decrease viral spread. Outpatient service areas should be restricted only to patients who require particular psychotropic medicines (*e.g*., need administration of medication at the institute) [84-86]. Appointments can be postponed for patients who are clinically stable and prefer telemedicine [86].

Shifting from traditional care to telepsychiatry can be useful in patients' follow-up, psychological treatment, and consultation with the caregiver. Due to the nature of the cognitive decline in psychiatric patients, these patients should be called *via* telephone by their mental staff to educate them about essential self-care during the pandemic [84-89]. Other communication methods that can be used for the delivery of mental health services include video conferencing, online forums, smartphone apps, text messaging, and e-mail [90-92]. Shifting outpatient and liaison services to telepsychiatry have received excellent feedback from patients, psychiatrists, and psychologists [93].

For patients with substance use disorders, psychiatric institutions must continue to provide care for maintenance treatment. Forms of substance use that are crucial to monitor continuously are opioid use and alcohol use [81]. Due to the restricted sales of alcoholic beverages and the limited capacity to acquire substances, adequate preparation for severe substance withdrawal symptoms is necessary for this situation. Proactive programs (*e.g*. harm reduction and psychosocial services) for reduced detrimental effects from substance use should be provided [80].

### 5.3 Inpatient Setting and Residential Facilities

Tightening admission criteria, shortening the length of hospital stays, and restricting access to hospital areas for newly admitted patients are strategies that have been used [21].

Infectious units or isolation wards in psychiatric hospitals can prevent the spread of SARS-CoV-2 from infected psychiatric patients to other patients [21]. When a patient manifests a viral infection symptom, he/she should be managed in a specific area. If patient isolation is not possible for any reason, the patient’s symptoms should be reported immediately to senior management and treated as an emergency [94].

Individual therapy can be provided while maintaining an appropriate interpersonal distance [86]. Some ward group activities (*e.g*., family meetings) should be suspended. Some activities (*e.g*., mindfulness, relaxation, and exercise) can be continued while everyone involved maintains a two-meter distance and wears masks [95, 96]. Notice boards, written communication, reduced group sizes, or individual meetings can help maintain excellent communication between staff and patients.

Face-to-face visits should be restricted to one hour per day. Only the close relatives of patients should be allowed to visit, and patients should be limited to one visitor at a time. The staff can provide video conferences for patient-family communication [80].

### 5.4 Community Psychiatry

Home visits are a way to monitor patients’ symptoms and adherence. Patients who need home visits include older adults, neglected patients, patients with cognitive impairment, and patients with intellectual disabilities [80]. Using mobile health or telehealth can help psychiatric patients with illness self-management and promote adherence, education, and symptom tracking [97].

Psychiatric drugs should be distributed to community hospitals, and community networks should be created to increase services. Drug delivery services should be provided for patients who have stable symptoms or are in disease remission. Long-acting medication can be used to maintain pharmacological treatment [80]. A way to minimize the rate of relapse is to prescribe large amounts of psychotropic drugs. It should be ensured that patients have an adequate amount of medicines for constant use [98].

Patients should be continuously informed about the situation (*e.g*., curfews) and provided with knowledge *e.g*., necessary hygiene recommendations. Education will help patients decreased the risk of severe COVID-19 outcomes, especially for psychiatric patients who also have a greater risk of COVID-19 mortality (*e.g*., those with hypertension, diabetes, chronic obstructive pulmonary disease, and coronary heart disease comorbidity) [84].

### 5.5 Mental Health Workers

Physical contact among psychiatric staff should be decreased by shifting regular staff meetings to virtual meetings and communicating mainly *via* telephone, e-mail, and instant messaging applications [93]. Unnecessary meetings should be cancelled [94]. Appropriate work shifts and regular breaks should be implemented to improve work efficiency and prevent burnout [8, 99-102].

Organizations should provide food, drink, and alternative accommodations for healthcare workers. Whenever there is a change in standards of practice, healthcare workers should be clearly informed of what has changed and why. Team leaders should recognize feelings of fear and anger in staff members, reactions to stress should be normalized, and support resources (*e.g*. sessions with psychologists/psychiatrists, online counseling should be provided for those who need it) [103]. Team leaders should actively monitor for moral injury, which is psychological distress that results from actions that violate someone's moral or ethical code. Staff exhibiting avoidance behavior, which is a core symptom of moral injury, should be identified. Team leaders can help decrease moral injury by clarifying the rationale behind decisions being made [72].

Staff support is one of the protective factors against adverse psychosocial effects [8, 104, 105]. Support programs should employ the following strategies. Training programs should be introduced for healthcare workers to help them identify and respond to psychological problems [106, 107]. In addition, healthcare workers should be given opportunities to reflect on their stress [108, 109] to help them normalize stress and intense emotions. Senior staff should be available for the staff to consult [110]. Guidelines for frontline staff to assess the authorities in a problematic situation should be available [94]. Around-the-clock telemental health services for healthcare professionals are useful [111].

## CONCLUSION

The COVID-19 outbreak has shaken the world and affected several aspects of human life. Specifically, patients with psychiatric problems are vulnerable to the crisis, which can exacerbate their conditions, with outcomes ranging from depression to suicide. Mental health professionals also experience both direct and indirect effects of COVID-19.

Therefore, mental health sectors should adapt the care they provide to cover a wide range of services and enhance the quality of care. Essential psychiatry care in emergency settings should be continued, but psychiatrists should limit direct contact with patients. Use telecommunication instead of face-to-face assessment in daily practice and preserve the standard of care. Keep patients informed about self-care during the outbreak. Drug delivery, distribution, and long-acting medication should be implemented. Mental health workers should be sufficiently supported.

There are some limitations to using this guide in clinical practice. Some facilities lack appropriate technologies to apply telecommunication. Some patients also have limited access to facilities or equipment for telecommunication. Psychiatrists may hesitate to utilize drug delivery services due to the risk of drug misuse. For the future outlook, we suppose that this study may help the mental health professionals prepared for the next outbreak of contagious disease. The direct impacts of COVID-19 on neuropsychiatric aspects should be studied further.

## Figures and Tables

**Appendix Table 1:** Impact on psychiatric patients and mental health professionals

Subject	Impact
Patients with psychosis	▪ Loss to follow-up ▪ Avoiding proper treatment ▪ More susceptible to infection ▪ More likely to become spreaders
Patients with depression	▪ Depression could be precipitated due to o Increasing stress o Disrupting normal routines and recreational activities o Social distancing o Decreased physical activity ▪ Anxiety about economic problem ▪ More prone to viral infection due to suppressed immunological function
Patients with suicidality	▪ Increasing rate of death by suicide due to o Unemployment o Social change o Economic recession o Financial problems
Mental health professionals	▪ Depression ▪ Anxiety ▪ Insomnia ▪ Posttraumatic stress disorder ▪ Burnout ▪ Moral injury ▪ Reduced workforce in psychiatric hospitals

**Appendix Table 2:** Management and care of psychiatric patients

Setting	Management
Emergency care	▪ Continue availability for the life-threatening condition. ▪ Psychiatrists use telephone consultation. ▪ Psychiatrists practice social distancing. ▪ Recognize neuropsychiatric symptoms of COVID-19.
Outpatient setting	▪ Decrease visits per day. ▪ Use telemedicine and postpone appointments for stable patients. ▪ Call patients by phone to educate them about self-care. ▪ Continue to provide maintenance treatment for patients with substance abuse. ▪ Prepare for substance withdrawal patients.
Inpatient setting and residential facilities	▪ Tighten admission criteria. ▪ Prepare restricting area for newly admitted patients. ▪ Establish infectious units or isolation ward. ▪ Suspend some group activities. ▪ Use notice boards for communication. ▪ Restrict visitors to close relatives, one visitor at a time. ▪ Provide video calls for patients and families.
Community psychiatry	▪ Use telepsychiatry. ▪ Use drug delivery services. ▪ Distribute drugs to community hospitals. ▪ Use long-acting medications. ▪ Inform patients about COVID-19.
Mental health workers	▪ Cancel the unnecessary meeting. ▪ Use telephone, e-mail, and instant messaging applications. ▪ Organization provides food, drinks, and accommodation. ▪ Inform staff members when there is a change in standard practice. ▪ Team leaders provide moral support. ▪ Provide support programs. ▪ Senior staff should be available. ▪ Have guidelines for problematic situations. ▪ Have around-the-clock telemental health services for staff members.
